# Implementation and evaluation of a training program as part of the Cooperative Biological Engagement Program in Azerbaijan

**DOI:** 10.3389/fpubh.2015.00228

**Published:** 2015-10-09

**Authors:** April Johnson, Gulshan Akhundova, Saida Aliyeva, Lisa Strelow

**Affiliations:** ^1^Signature Science, LLC.Baku, Azerbaijan; ^2^Technology Management CompanyBaku, Azerbaijan; ^3^Civilian Research Development FoundationBaku, Azerbaijan; ^4^Bechtel National Inc.Baku, Azerbaijan

**Keywords:** Cooperative Biological Engagement Program, training, Azerbaijan, Ministry of Health, Ministry of Agriculture

## Abstract

A training program for animal and human health professionals has been implemented in Azerbaijan through a joint agreement between the United States Defense Threat Reduction Agency and the Government of Azerbaijan. The training program is administered as part of the Cooperative Biological Engagement Program, and targets key employees in Azerbaijan's disease surveillance system including physicians, veterinarians, epidemiologists, and laboratory personnel. Training is aimed at improving detection, diagnosis, and response to especially dangerous pathogens (EDPs), although the techniques and methodologies can be applied to other pathogens and diseases of concern. Biosafety and biosecurity training is provided to all trainees within the program. Prior to 2014, a variety of international agencies and organizations provided training, which resulted in gaps related to lack of coordination of training materials and content. In 2014 a new training program was implemented in order to address those gaps. This paper provides an overview of the Cooperative Biological Engagement Program training program in Azerbaijan, a description of how the program fits into existing national training infrastructure, and an evaluation of the new program's effectiveness to date. Long-term sustainability of the program is also discussed.

## Introduction and program background

The Cooperative Biological Engagement Program (CBEP), under the US Defense Threat Reduction Agency's broader Cooperative Threat Reduction program, works with approximately 20 countries around the world to combat biological threats [1]. Objectives of the program are to prevent the proliferation of biological weapons; consolidate and secure collections of dangerous pathogens in central live microorganism repositories; strengthen biosafety and biosecurity of laboratory facilities; and improve partner nations' ability to detect, diagnose, report, and respond to outbreaks of disease caused by especially dangerous pathogens (EDPs). EDPs are infectious agents with the potential for use as biological weapons that may result in significant harm to people or animals.

The CBEP was first started to be implemented in Azerbaijan in 2007. Bechtel National, Inc. (BNI) has been working as the integrating contractor there since 2011. The CBEP works primarily with the Ministry of Health (MoH) and State Veterinary Control Service (SVCS) within the Ministry of Agriculture. MoH and SVCS administer the national surveillance systems for human and animal diseases, respectively, and are therefore the main recipients of program support as the implementing agencies. CBEP funded the construction or renovation of biosafety level 2 (BSL-2) laboratories throughout the country—five for human health [four regional and one Republican (national) level] and six for animal health (five regional and one Republican level). Modern diagnostic equipment was provided for these twelve laboratories (one additional laboratory for Ministry of Defense), as was training on facility and equipment maintenance, training on equipment use, and Biosafety and Biosecurity (BS&S) training. The MoH is constructing a combination BSL-2/BSL-3 Republican-level laboratory in Baku with their funds that will serve as the Central Reference Laboratory for Azerbaijan. CBEP is assisting with construction oversight and training at this facility. Construction completion is expected in 2016.

Several EDPs are endemic in Azerbaijan. There are approximately 300–500 human cases of brucellosis reported annually [2, 3]. Anthrax cases continue to be reported, in spite of livestock vaccination campaigns [4]. In a recent study, human volunteers were seropositive for tularemia [5]. One study found that not all people who are ill will seek healthcare, so reported cases of EDPs may be underestimated, as the country relies on passive surveillance from the healthcare system for disease detection [6]. Strengthening recognition of EDP cases by healthcare workers and animal health workers becomes even more important for detecting outbreaks if few people seek medical care for infectious diseases. The CBEP provides training aimed at improving rapid recognition, diagnosis, reporting, and response among key members of the Azerbaijani surveillance systems, including infectious disease specialists, human and animal clinicians, epidemiologists, and laboratory staff.

CBEP training in Azerbaijan has been delivered in two phases. From 2007 to 2010, training in clinical recognition of diseases, epidemiology, and laboratory diagnostics was provided by several agencies and organizations including the US Army Medical Research Institute of Infectious Disease, Walter Reed Army Institute of Research, US Centers for Disease Control and Prevention, UK Public Health England, and UK Animal and Plant Health Agency. The integrating contractor Black and Veatch introduced (and continues to train on) the Electronic Integrated Disease Surveillance System, which is an electronic disease reporting system integrated into the MoH and Ministry of Agriculture disease surveillance systems.

In 2011, BNI conducted a training gap analysis to establish the baseline knowledge of Government of Azerbaijan staff and the broader facility capabilities associated with CBEP in Azerbaijan. Gaps between training provided through 2010 and the competencies necessary to fulfill CBEP goals and ensure program sustainability were identified and taken into consideration when developing the new training program. Among other gaps, the analysis identified inconsistencies in materials, as well as topics that were not rigorously addressed in the original program, such as sample packaging and transport. A new training program aimed at addressing identified gaps was implemented in 2014. In the context of this new CBEP training program, BNI is delivering clinical disease recognition, epidemiology, and laboratory training in partnership with Government of Azerbaijan trainers, and continuing to provide BS&S training.

This paper describes the implementation and evaluation of the new training program developed by BNI in collaboration with the Defense Threat Reduction Agency and the Government of Azerbaijan from 2014 to 2015. Data from trainings conducted between September 2014 and April 2015 and annual BS&S trainings conducted in 2013–2014 are included in the evaluation of the program.

## Methods

### Implementation of training

The CBEP training program was developed by BNI in collaboration with the governments of the US and Azerbaijan. The following disciplines, targeting both human and animal health workers, were chosen for inclusion: Clinical Recognition of Infections Caused by EDPs, Epidemiology, and three Laboratory disciplines (Bacteriology, Serology, and PCR). With the exception of Clinical trainings, Basic and Advanced level courses are being taught for each discipline, based on a needs assessment to identify appropriate trainees for the advanced courses. Since both ministries agreed to a One Health approach to Epidemiology training, the trainee pool for this discipline comprises an equal number of participants from the MoH and the SVCS. The Epidemiology courses include a mixture of joint lectures and exercises, as well as separate Ministry-specific breakout sessions. BNI has provided annual biosafety and biosecurity (BS&S) training for laboratory staff since 2013, and will deliver Biosafety Officer training to designated trainees. Together with Black & Veatch, BNI will also provide BS&S training to Central Reference Laboratory staff.

To ensure that all Azerbaijani training needs were taken into account in the CBEP training program, training sub-working groups (TSWGs) were established with both Ministries. These groups met twice monthly between April and September 2014. The TSWGs consisted of four individuals from SVCS and nine individuals from MoH. The working groups identified subject matter experts for each discipline (Clinical, Epidemiology, Bacteriology, PCR, Serology, and BS&S) to assist with material development and review. Using pre-qualification criteria that were developed for each subject discipline, the working groups also identified the trainees who would most benefit from the training and represent the broader target audience geographically.

#### Training materials

Training materials are based on modules previously developed by CBEP subject matter experts and provided to BNI as Government-furnished information adapted for use in Azerbaijan. BNI also developed training materials when previously developed materials were not available. Subject matter experts identified by the TSWGs reviewed the training materials, adapting them and providing examples specific to Azerbaijan as needed. All materials were approved by the Defense Threat Reduction Agency, MoH, and SVCS before training implementation. Efforts were made to use the most up-to-date information available in the English language scientific literature, and to reference international standards where applicable. Trainees are encouraged to report inaccurate translations or other errors noted during training on comment forms, so as to improve materials for future delivery. Relevant consensus changes suggested by trainees and trainers will continue to be incorporated into the materials throughout delivery of the courses.

All trainees are provided with a hard copy of the training materials to use during the training and will receive electronic versions of the materials once all training is delivered and the materials are finalized (i.e., no further translation changes are made during course delivery). At the request of MoH, training materials (slides and slide notes, plus additional background materials) will also be compiled into bound booklets to serve as a reference for future trainers.

#### Trainers

Prior to training implementation in September 2014, the MoH and SVCS identified co-trainers to administer training alongside BNI. The training program will transition entirely to the Government of Azerbaijan in January 2016, so it is important to help develop the Government of Azerbaijan trainers quickly. All Government of Azerbaijan co-trainers participated in an Adult Learning Principles course conducted by BNI to improve their training skills and to ensure a consistent approach to training delivery among all trainers. After observing one course delivered exclusively by a BNI expatriate trainer, these Government of Azerbaijan trainers co-deliver an increasing proportion of course content with each subsequent training event until they are training 100% of the course. This occurs during the last scheduled course, if not sooner. Individual co-trainers will have covered every topic in the discipline by the end of the scheduled trainings, and will continue to deliver this training beyond CBEP's period of engagement, ultimately sustaining the training program in Azerbaijan for years to come.

Training events are delivered by a team comprising one BNI expatriate trainer, 1–3 local national BNI training facilitators, and 1–4 Government of Azerbaijan co-trainers. The total number of trainers for any given event depends on the course discipline and number of trainees. Prior to each event, the training teams meet to allocate assignments, review course material as needed, and to clarify any logistical issues. During the training, teams meet as necessary to address any training or logistical issues and to discuss how the training delivery and training materials can be improved.

Training conducted by expatriate trainers requires interpretation from English; training conducted by local national facilitators and Government of Azerbaijan trainers is conducted in Azerbaijani. The MoH-identified co-trainers are authorized by the Azerbaijani State Doctors Improvement Institute (SDII) to provide continuing education course credit to doctoral level staff who successfully pass CBEP post-training tests. MoH co-trainers who are registered with the Baku Base Medical College #2 (BBMC2) are likewise authorized to provide continuing education course credit to laboratory technician level staff who successfully pass CBEP post-training tests.

#### Trainee identification

Trainees were selected by the TSWGs at the start of the program, with changes made as necessary based on trainee availability. To cover the widest geographic area in a limited amount of time, trainees from each rayon (region) were chosen for each discipline (clinical human, clinical vet, human epidemiology, and veterinary epidemiology) based on their job duties; more than one individual was targeted in each rayon to provide redundancy in the event that a trainee leaves the system. Four individuals per laboratory were targeted for Basic training in each discipline; from this pool, two trainees will be selected for advanced training based on individual job duties and performance during the Basic level course. Trainees are expected to train eligible participants in their rayons or laboratories who did not participate in CBEP training events.

Pre-qualification criteria were developed to identify the most appropriate trainees. For example, Epidemiology trainees must be (1) employed by the relevant ministry, (2) responsible for conducting epidemiological investigations of EDP cases, (3) responsible for data entry into the Electronic Integrated Disease Surveillance System, (4) responsible for advising on and implementing prevention and control measures against EDPs for the rayon, (5) 60 years of age or less, and (6) willing to train others. Similarly, Clinical trainees must be (1) employed by the relevant ministry, (2) responsible for diagnosing cases of disease suspected to be caused by EDPs, (3) 60 years of age or less, and (4) willing to train others.

#### Training delivery

A comprehensive training schedule covering 15 months (September 2014–November 2015) was developed by BNI in collaboration with the MoH and SVCS. Training events for the laboratory disciplines are held at the 12 CBEP-engaged laboratories. This includes five regional veterinary laboratories (Zonal Veterinary Laboratories), four regional human health laboratories (Anti-Plague Division Laboratories), and three laboratories in Baku (the Republican Anti-Plague Station, Republican Veterinary Laboratory, and the Ministry of Defense Center for Sanitary-Epidemiological Control Laboratory). Clinical and Epidemiology trainings are held in conference rooms in hotels in five cities throughout Azerbaijan (Baku, Ganja, Guba, Gakh, and Lankaran). Trainees who live more than 1 h from the site are provided with lodging at the hotel where the training will take place. Laboratory course size is limited to approximately four trainees for Basic and two trainees for Advanced courses. Clinical courses are limited to approximately 20 trainees. Epidemiology trainings are conducted for approximately 16 trainees at a time, half of whom are human health epidemiologists and half of whom are veterinary epidemiologists. Annual BS&S training takes place at each of the 12 laboratories for an average of 10 trainees per laboratory.

### Evaluation of training

The most widely used method for evaluating training programs is “The Four Levels of Learning Evaluation” developed by Kirkpatrick in 1959 and later revised [7]. The four levels of evaluation are *Reaction, Learning, Behavior*, and *Results*. *Reaction* was assessed by administration of anonymous trainee evaluation forms that were filled out daily during training events. *Learning* was assessed by way of pre- and post-training test scores. *Behavior* and *Results* require long-term evaluation and are further addressed in the Discussion Section.

Complete data is available for courses administered by BNI between September 2014 and April 2015 for the Clinical, Epidemiology, and Laboratory disciplines, as well as annual BS&S training events conducted in 2013 and 2014.

#### Reaction

The first level of learning represents the satisfaction of the trainees with the training. This was evaluated by means of anonymous trainee evaluation forms. Standardized questionnaires were developed and are administered to trainees at the end of each training day to solicit their opinions on the content of the training, quality of the training, and knowledge and skill levels of the trainers on a five-point scale (*Poor*/*Below Average*/*Met Expectations*/*Above Average/Excellent*). Three-point scales (*Enough*/*Just Right*/*Not Enough, Basic/Appropriate/Too Advanced*, and *Yes/Not Sure/No*) are used to determine if allotted time, content, and practical exercises were sufficient; if the material was too basic or advanced; and if the material is relevant to their current positions. Open-ended questions provide an opportunity for trainees to comment on the lectures they liked most or least, and to seek input on how to improve the training. Input was sought daily while the information was fresh, rather than waiting until the end of the event, when trainees would be less likely to recall earlier trainers and lectures. Input from each day was summarized and compiled for a complete evaluation of each event. The data presented herein is derived from overall summaries of trainee evaluations from Clinical, Epidemiology, and Laboratory trainings conducted between September 2014 and April 2015.

#### Learning

Pre- and post-training tests were used to evaluate the knowledge gained during a given course. A pre-training test was administered on the first day of the course to gauge baseline knowledge, and the same test was administered at the end of the course to evaluate knowledge gain. Tests are usually 25 questions in length (range 15–30 questions) and are comprised of multiple choice and some true/false questions. Data from trainees who did not complete both the pre- and post-training tests are not included in the summary.

## Results

### Implementation of training

Planning for the BNI training program began in April 2014. A total of 74 training events (targeting 584 total trainees) were planned from September 2014 to December 2015 in the Clinical Recognition of Infections caused by EDPs, Epidemiology, Bacteriology, Serology, and PCR disciplines. EDPs covered in the training program are listed in Table 1. Between September 2014 and April 2015, 46 training events were conducted for 386 trainees. From 2013 to 2014, 24 BS&S annual refresher training events were held for a total of 196 participants.

**Table 1:** **Especially Dangerous Pathogens (EDPs) covered in the CBEP training program**.

**Human health**	**Animal health**	**Zoonoses**
*Yersinia pestis* (plague) *Francisella tularensis* (tularemia) Crimean-Congo hemorrhagic fever virus Tick-borne encephalitis virus Smallpox virus *Clostridium botulinum*	Capripox virus Newcastle disease virus African swine fever virus Classical swine fever virus Foot and mouth disease virus *Burkholderia mallei* (glanders) Rinderpest virus Peste des Petits Ruminants virus	Avian influenza virus *Brucella* species (brucellosis) *Bacillus anthracis* (anthrax) *Coxiella burnetii* (Q fever)

The list of co-trainers identified by the Government of Azerbaijan evolved over the course of the program, as competing responsibilities arose or trainers left their job posts. As new trainers were identified, additional courses in Adult Learning Principles were provided to ensure that all trainers were equipped to provide training, and all materials were provided well in advance of training to allow trainers to gain familiarity with the materials. A total of six events in Adult Learning Principles were provided to small groups of Government of Azerbaijan co-trainers. Refer to Table 2 for the numbers of Government of Azerbaijan trainers in the program.

**Table 2:** **Total number of MoH and SVCS co-trainers active in the program as of April 2015**.

**Categories**	**No. of MoH trainers**	**No. of SVCS trainers**
Clinical recognition	2	2
Epidemiology	3	4
Bacteriology	3	3
Serology	2	3
PCR	3	3
Biosafety and biosecurity	2	2
Total	15	17

### Evaluation of training

#### Reaction

**Figure 1:**
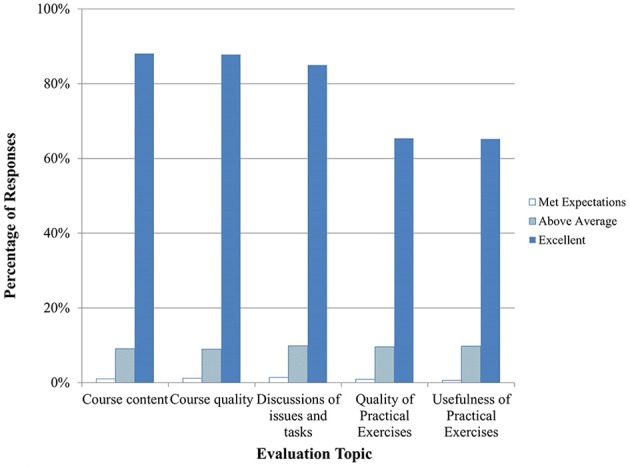
**Clinical training evaluation by trainees (*n* = 873–926 total daily responses for each question)**. Note that no responses were marked as poor or below average so those categories are not shown.

**Figure 2:**
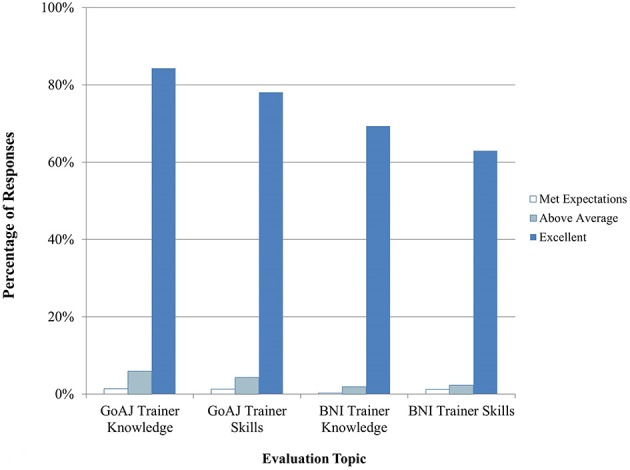
**Trainee feedback on Clinical trainer knowledge and skills (*n* = 909–1687 total daily responses for each question)**. The number of N/A responses to these questions is not shown.

**Figure 3:**
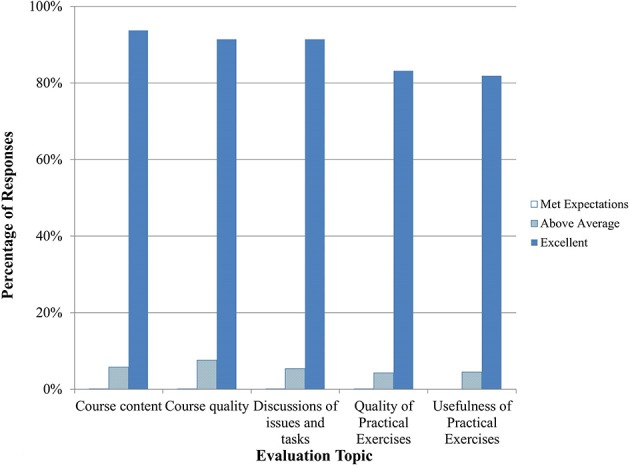
**Epidemiology training evaluation by trainees (*n* = 1018–1107 total daily responses for each question)**. Note that no responses were marked as poor or below average so those categories are not shown.

**Figure 4:**
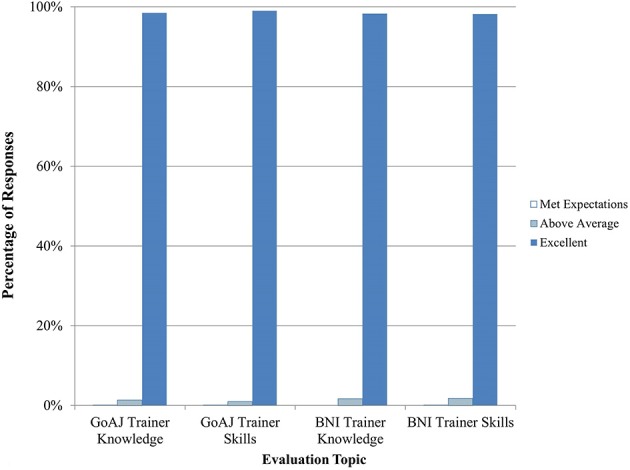
**Trainee feedback on Epidemiology trainer knowledge and skills (*n* = 1810–2011 total daily responses for each question)**. The number of N/A responses to these questions is not shown.

**Figure 5:**
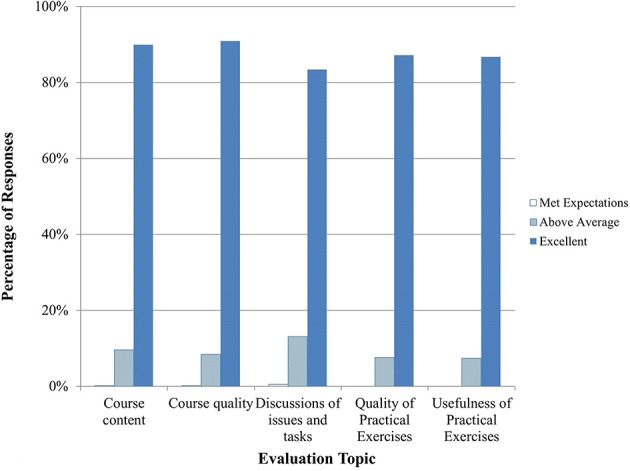
**Laboratory training evaluation by trainees (*n* = 421–427 total daily responses for each question)**. Note that no responses were marked as poor or below average so those categories are not shown.

**Figure 6:**
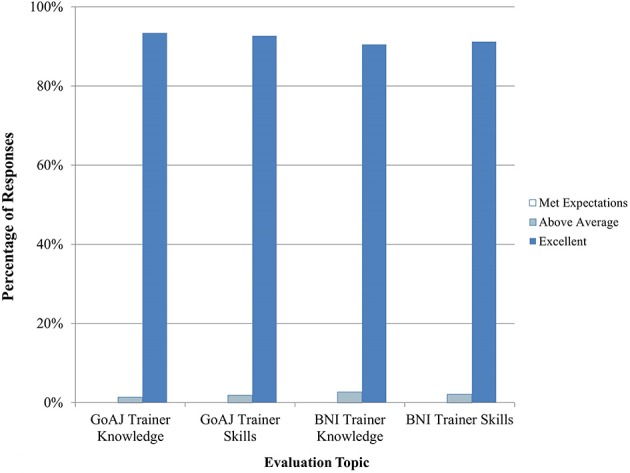
**Trainee feedback on Laboratory trainer knowledge and skills (*n* = 262–485 total daily responses for each question)**. The number of N/A responses to these questions is not shown.

Since evaluation forms were requested to be filled out daily, response rates tended to be fairly high. However, trainees did not always answer every question, as noted by the variable numbers of responses in Figures 1–**6**.

Overall, approximately 97% of trainees felt that the training covered topics that would be useful for them in their daily work, and 78% felt that the level of the material was appropriate (neither too advanced nor too basic). About 80% felt there was sufficient time to cover the material being presented, and that the amount of content and practical exercises was sufficient. Results of questions on course content and quality and trainee perceptions of the knowledge and skill levels of both BNI and Government of Azerbaijan trainers can be found in Figures 1–**6**.

##### Clinical training

Between September 2014 and April 2015, 13 courses in Clinical Recognition of Infections Caused by EDPs were delivered, seven for physicians and six for veterinarians. A summary of trainee feedback is shown in Figures 1, 2. Over 80% of trainees responded that the course content, quality, and discussions were excellent. 65% of trainees felt that the practical exercises were excellent in quality and usefulness, while about 24% responded Not Applicable (N/A). Hands-on practical exercises for Clinical courses are challenging to implement since human patients cannot be used and animals cannot be brought to training sites; therefore, practical exercises for this discipline are limited to case studies and scenario exercises.

##### Epidemiology

Seven courses in Epidemiology were conducted between September 2014 and April 2015 for mixed audiences of human and veterinary epidemiologists. Practical exercises in this course included case studies and exercises. Overall, trainees were satisfied with the course content and quality (Figure 3). They enjoyed the joint One Health approach and asked for additional lectures to be provided as joint trainings. Trainees were very satisfied with the knowledge and skills of both the BNI and Government of Azerbaijan trainers (Figure 4).

##### Laboratory training

Nineteen Laboratory training events were conducted between September 2014 and April 2015. Practical exercises were conducted to reinforce skills in pipetting, setting up and running assays, and interpreting results. About 87% of trainees rated the exercises as excellent in terms of quality and usefulness and 90% or more felt that the course content and quality and trainer knowledge and skills were excellent (Figures 5, 6).

#### Learning

Of 386 trainees, pre- and post-training test scores were available for 375 trainees (97%). Overall, post-training test scores were higher than pre-training scores (Table 3), indicating an increase in trainee knowledge. Due to the small class sizes, data from the MoH and SVCS trainees were combined for the Laboratory disciplines.

**Table 3:** **Median pre- and post-test scores for training events between September 2014 and April 2015, with 25–75% interquartile ranges**.

**Category**	**No. of courses**	**No. of trainees**	**No. Trainees with pre/post-test scores**	**Pre test % (Q1–Q3)**	**Post test % (Q1–3)**	**% increase (Q1–Q3)**
Clinical recognition of infections caused by EDPs (MoH)	7	106	100	47.5 (36 − 53)	77 (67 − 86)	30 (21 − 40)
Clinical recognition of infections caused by EDPs (SVCS)	6	97	97	47 (36 − 59)	75 (64 − 82)	25 (13 − 39)
Epidemiology (MoH)^*^	7	60	59	45.5 (36.5−53)	69 (59 − 78)	23 (15.5−28.5)
Epidemiology (SVCS)^*^	7	53	53	34.5 (30.5−41.5)	54.5 (47 − 67)	19.5 (9 − 29)
Basic PCR	5	19	17	32 (16 − 48)	80 (80 − 88)	40 (20 − 60)
Advanced PCR	4	10	8	40 (32 − 50)	73.5 (70 − 83)	28.5 (24−45.5)
Basic serology	4	20	20	27 (18−28.5)	87 (80 − 93)	66 (47 − 71)
Advanced serology	2	4	4	44.5 (41.5−48)	76 (61−88.5)	30.5 (13 − 46)
Basic bacteriology	2	10	10	50 (36 − 59)	70 (61−77.5)	17.5 (10 − 25)
Advanced bacteriology	2	7	7	55 (42.5−57.5)	85 (82.5−87.5)	30 (27.5−42.5)

* *Tests were administered each week of a 2-week course; scores represent an average of the two pre-training test and the two post-training test scores*.

##### Annual biosafety and biosecurity refresher training

**Figure 7:**
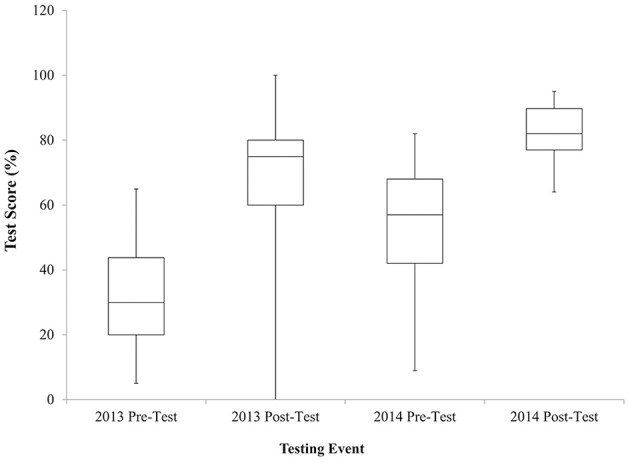
**Quartile values of median pre- and post-training test scores for Annual BS&S Refresher training events for 2013 and 2014**. Note that one individual in 2013 scored a 0 on the post-training test which was later determined to be due to an inability to read Azerbaijani. Following this result, a Russian translation was also made available, or the tests were read aloud to individuals who could not read Azerbaijani. Excluding this individual, the lowest score on a post-training test was 30%.

From 2013 to 2014, 196 total trainees from the twelve CBEP-engaged laboratories participated in annual BS&S refresher training (97 in 2013 and 99 in 2014). The median scores and interquartile ranges from pre- and post-training tests are shown in Figure 7. The median pre-training test score in 2013 was 30% and the median post-training test score was 75%. In 2014, the test scores increased from a median of 57% on the pre-training test to 82% on the post-training test.

## Discussion

Since the beginning of CBEP engagement in Azerbaijan, 1710 individual human and animal healthcare workers and laboratory staff have received training. Since implementation of the new training program in September 2014, the program has trained 32 Government of Azerbaijan trainers, including at least two trainers for each discipline from each ministry. Between September 2014 and April 2015, 386 MoH, and SVCS staff members were trained in Clinical Disease Recognition, Epidemiology, and Laboratory disciplines. An additional 400 trainees are expected to have received training by the end of 2015.

### Evaluation of the training program

Trainees rated the training program content and quality highly on anonymous feedback forms and the majority felt the information trained would be useful in their work. Their responses to open-ended questions showed which topics were of most interest and use to the trainees. Many respondents asked for additional trainings in the future.

Overall, median scores for pre-training tests increased for each discipline. Median Epidemiology test scores are uniformly lower than those in other disciplines, and especially for veterinary staff; the median Epidemiology post-training test scores fell below CBEP's threshold for a passing grade (70%). During the training events, it became evident that the epidemiology content was new to most of the trainees and that the concepts discussed were not routinely used in daily activities. As a result, further discussions were held with both ministries to identify the most effective approach to follow-on training.

Annual BS&S training pre-training test scores increased from 30% in 2013 to 57% in 2014. Fifty-three percent of those trained in 2014 were also trained in 2013. This increase in scores from 2013 to 2014 suggests that there might be some residual knowledge from the previous years' training or shared knowledge by trained staff to new staff; data from annual BS&S training events in 2015 will be analyzed to see if pre-training test scores continue to rise within this trainee pool.

Training programs should result in behavior change and positive outcomes, which are the third and fourth levels of Kirkpatrick's training evaluation model and are more difficult to assess than the first and second levels [7]. There are six local national BNI staff members who visit the twelve CBEP-engaged laboratories twice a month to observe regular laboratory practices and behaviors, to provide mentorship as necessary, and to administer proficiency testing in Laboratory disciplines. A laboratory assessment tool was recently implemented to quantitatively assess performance over time, but only at the laboratory level and not at the individual trainee level. The PCR proficiency testing program was re-implemented by BNI in October 2014; the proficiency testing programs in Bacteriology and Serology are scheduled to begin in June 2015. The proficiency testing program allows ongoing assessments of individual trainee performance and behavior in the areas of BS&S and laboratory diagnostic testing. Assessing behavior change among Clinical and Epidemiology trainees is more challenging, as BNI interacts less frequently with those individuals and they are more geographically dispersed. BNI plans to administer post-training questionnaires and tests to evaluate learning retention, and to assess attitudes and practices in these trainee pools.

One goal of the CBEP training program is a surveillance system that can rapidly and effectively recognize, report, diagnose, and respond to EDPs; adherence to appropriate BS&S practices is another critical result. EDP outbreaks occur only sporadically—it is therefore problematic to measure success of such outcomes in a natural setting. However, several cases of EDPs have been diagnosed by Government of Azerbaijan personnel at the CBEP-engaged laboratories using techniques introduced by CBEP. In addition, the Defense Threat Reduction Agency assessed a vertical slice of the human and animal disease surveillance systems by means of an operational demonstration in March 2014. In this exercise, a hypothetical anthrax outbreak scenario was presented to veterinary and human clinicians and epidemiologists to assess how they would respond. The assessment included their ability to gather appropriate physical exam histories; create a differential diagnosis; collect appropriate diagnostic samples in a safe manner; package and transport the samples; and to correctly report the disease in the Electronic Integrated Disease Surveillance System. To demonstrate diagnostic and biosafety practices, laboratory staff were assessed on how they tested non-pathogenic samples of the specific disease agent using CBEP-trained techniques. Overall, although both the human and animal health systems were found to be acceptable, areas for additional training were identified and incorporated into the current training program or are being addressed via mentorship visits.

### Training challenges

There are several obstacles to the long-term success of the CBEP training program in Azerbaijan including an aging workforce, a limited pool of qualified trainers, a lack of a post-graduate training entity for SVCS staff, and limited resources designated for EDP training.

As salaries are low in comparison with non-state sectors and recruitment/retention of newly graduated or young staff is consequently difficult, resulting in a workforce with many staff members close to retirement age. An initial cutoff of 50 years of age was proposed for trainees in Clinical Recognition and Epidemiology disciplines, but neither ministry felt that they could identify sufficient numbers of trainees under 50 years of age. The gap analysis conducted in 2011 found that 42% of 110 human and veterinary facility staff who volunteered their age was over the age of 50. As existing personnel retire and new personnel are hired, ongoing training will be necessary to bring new staff up to speed.

The turnover of Government of Azerbaijan trainers in the training program observed to date presents an ongoing risk to training continuity if the trend continues. There is a small pool of qualified trainers, many of whom have competing responsibilities as part of their routine work duties. Of 27 trainers initially identified by MoH in fall of 2014, only 13 are still trainers; two new trainers have also been added. The SVCS has likewise made several changes to the trainer list: of 20 originally trainers, five were removed and three were added, one of whom was subsequently removed. Such ongoing changes reduce the amount of time available for newly identified trainers to gain familiarity with the materials and build experience in delivering training before the transition of the CBEP training program to the Government of Azerbaijan.

At present, the SVCS has neither designated an institute to provide continuing education for veterinarians, nor mandated a certain amount of regular continuing education. SVCS epidemiologists and veterinarians are only trained during veterinary school. Veterinary epidemiologists receive most of their training on-the-job, and neither group receives any formal continued education (unlike the MoH doctors, who must receive training every five years at the SDII). This lack of a formal post-graduate training program means that veterinarians are not receiving refresher training or keeping up with new advancements in the field. Additional efforts will be needed to ensure that the CBEP-provided EDP training courses continue.

Resources for training are limited, and therefore additional funding must be requested for ongoing training to be sustained by each Ministry starting in 2016. BNI is working with the ministries to provide budget estimates for the training program in order to ensure that sufficient funds are requested.

### Future CBEP training

The MoH has identified two training entities to continue the CBEP trainings. The SDII provides ongoing continuing education to physicians, epidemiologists, and laboratory doctor level staff, and serves as the licensing agency for human healthcare workers. Doctors receive training and take the licensing examination every 5 years. As of September 2014, all MoH trainees in CBEP disciplines (see Table 3) who receive a post-training test score of 50% or more receive SDII credit. MoH plans to continue training CBEP-provided courses through SDII with the use of MoH trainers starting in September 2015. Similarly, the BBMC2 serves as the post-graduate training entity for MoH laboratory technicians. One MoH trainer in each discipline is registered with BBMC2, which allows them to provide course credit to laboratory technician trainees. These trainers will continue to provide training to these individuals at the Anti-Plague Division regional laboratories since the BBMC2 does not have the same diagnostic equipment as the CBEP-engaged laboratories. Training at BBMC2 is traditionally theoretical and not practical, so training at the laboratories will allow technicians to continue to receive practical hands-on training in EDP diagnostic testing.

As previously discussed, the SVCS does not have a post-graduate training institute, although the issue has been raised with the Minister of Agriculture. In the meantime, the SVCS plans to request funding to start conducting training using the CBEP-trained trainers and possibly top-performing trainees from the training events conducted during the current CBEP training program. Without a designated training institute, it will be more challenging to continue the training program after transition of the training program to SVCS.

All relevant CBEP training materials and records will be provided to each ministry. Training materials will be finalized in conjunction with Government of Azerbaijan trainers to ensure that materials are comprehensive and sufficiently detailed that newly identified trainers can easily pick up the materials to conduct training. Azerbaijan-specific and regional examples are being included to ensure that the materials are relevant.

An electronic training database will also be shared with each ministry. The database outlines all the trainings conducted to date, the trainees who participated, and pre- and post-training test scores. This will not only pertain to the recent CBEP training program, but to all trainings conducted to date by CBEP collaborators and integrating contractors. Training records will help the Government of Azerbaijan to identify participants for future training events and to record progress.

## Conclusion

Overall the CBEP training program has been successful, as evidenced by positive trainee feedback, Government of Azerbaijan's desire to continue training CBEP courses in the future, and MoH's willingness to integrate CBEP training into current national training curricula. Improvements in pre- and post-training test scores also indicate that training has been effective. Ongoing evaluation will help to identify changes in behavior and in the ability of the human and animal disease detection and response systems to recognize and respond to EDP outbreaks. Continued training will ensure that newly hired personnel are integrated into the surveillance system, that Government of Azerbaijan trainers maintain their training skills, and that those engaged in surveillance activities maintain their ability to respond to EDPs in the future.

### Conflict of interest statement

The authors declare that the research was conducted in the absence of any commercial or financial relationships that could be construed as a potential conflict of interest.
